# Genetic mutations in influenza H3N2 viruses from a 2012 epidemic in Southern China

**DOI:** 10.1186/1743-422X-10-345

**Published:** 2013-11-26

**Authors:** Jing Zhong, Lijun Liang, Ping Huang, Xiaolan Zhu, Lirong Zou, Shouyi Yu, Xin Zhang, Yonghui Zhang, Hanzhong Ni, Jin Yan

**Affiliations:** 1 Key Laboratory for Emergency Pathogen Detection, Guangdong Provincial Center for Disease Control and Prevention, Guangzhou 511430, China; 2 Department of Infectious Diseases, Guangzhou Chest Hospital, Guangzhou 510095, China; 3 Department of Epidemiology, School of Public Health, Sun Yat-Sen University, Guangzhou 510080, China; 4 Department of Epidemiology, School of Public Health and Tropic Medicine, Southern Medical University, Guangzhou 510515, China

**Keywords:** Influenza, H3N2, Phylogenetics, Mutation, Epidemic

## Abstract

**Background:**

An influenza H3N2 epidemic occurred throughout Southern China in 2012.

**Methods:**

We analyzed the hemagglutinin (HA) and neuraminidase (NA) genes of influenza H3N2 strains isolated between 2011–2012 from Guangdong. Mutation sites, evolutionary selection, antigenic sites, and N-glycosylation within these strains were analyzed.

**Results:**

The 2011–2012 Guangdong strains contained the HA-A214S, HA-V239I, HA-N328S, NA-L81P, and NA-D93G mutations, similar to those seen in the A/ Perth/16/2009 influenza strain. The HA-NSS_061–063_ and NNS_160–162_ glycosylation sites were prevalent among the 2011–2012 Guangdong strains but the NA-NRS_402–404_ site was deleted. Antigenically, there was a four-fold difference between A/Perth/16/2009 -like strains and the 2011–2012 Guangdong strains.

**Conclusion:**

Antigenic drift of the H3N2 subtype contributed to the occurrence of the Southern China influenza epidemic of 2012.

## Introduction

Influenza virus undergoes rapid evolution by both antigenic shift and antigenic drift and this presents a significant challenge for vaccine design to best match with viruses likely to circulate in the coming influenza season [1,2]. Since their emergence in 1968, influenza H3N2 viruses tend to be highly prevalent most years. The two dominant proteins in this particular virus strains are the H3 hemagglutinin (HA) and N2 neuraminidase (NA) surface glycoproteins [1,3]. Antigenic drift, also known as the result of positive selection, generally involves both of these proteins, resulting in evasion of the host immune response [4]. Many amino acid variations occur at the antibody binding sites, creating diversity in the HA and NA proteins and allowing them to evade host antibodies. However, these variations do not significantly alter the stereochemical or functional properties of HA and NA.

In Canada, antigenic drift was observed in 2008 when the A/Brisbane/10/2007 strain (the vaccine strain used in 2008–2009 and 2009–2010) mutated into the A/Perth/16/2009 strain (vaccine strain used in 2010–2011) [5]. Antigenic and molecular characterization of H3N2 viruses over those three seasons revealed that the number of HA mutations is important, along with the nature and location of key mutations, and likely plays a significant role in antigenic drift. In previous work, amino acid substitutions were seen in five antigenic regions of HA1 from 2007–2011 Guangdong (for short GD) isolates; in particular, regions B (N160K) and D (K174R/N). The K189E/N/Q and T228A mutations within the receptor-binding site (RBS) were present in the 2010 strains, affecting the antigenicity of HA1 [6]. The antigenicity of epidemic H3N2 isolates in 2011 differed from that of the A/Perth/16/2009 strain.

The evolution and epidemiology of influenza H3N2 viruses is partially related to global migration dynamics [7]. The evolution of H3N2 influenza over the past 10 years reflects the dynamics of a global metapopulation. According to the Center for Public Health Surveillance and Information Service of China [8], the number of influenza cases was 2.35-fold greater for January–June 2012 (74,151 cases) than for January–June 2011 (31,551 cases). Approximately 87% (365/418) of isolates were of the H3N2 subtype; these isolates were sourced from local epidemics and sporadic cases reported in Guangdong Province from March–June 2012. We analyzed HA and NA gene sequences of H3N2 viruses isolated from July 2011 to July 2012.

## Materials and methods

### Viruses and genes

**Figure 1:**
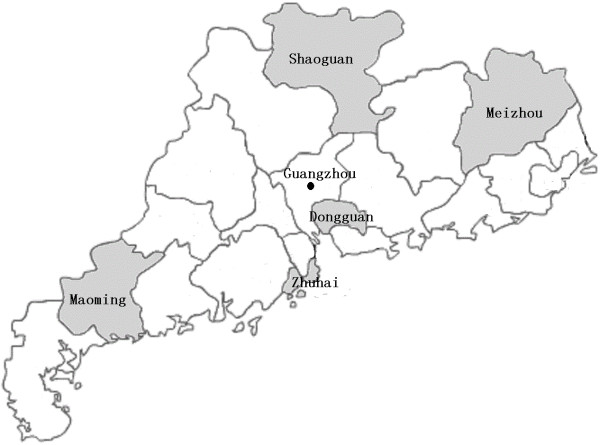
**Strains sampled from different cities in Guangdong.** Stains were isolated from the samples included three from Zhuhai, two from Shaoguan, three separately from Foshan, Maoming and Meizhou.

With assistance from the Guangdong Influenza Surveillance Network, H3N2 viruses from 2011–2012 were isolated in MDCK cell lines [6]. Viruses investigated in our study included four 2011 and four 2012 isolates (GenBank Accession Nos. CY125677–CY125692). Isolates from Zhuhai, Shaoguan, Foshan, Maoming and Meizhou are presented in Figure 1 and Table 1. Around 60 HA and 55 NA gene sequences were downloaded from the Influenza Virus Resource (http://www.ncbi.nlm.nih.gov/genomes/FLU/Database/nph-select.cgi?go=alignment), and included vaccine strains A/Wyoing/03/2003, A/California/ 07/2004, A/Wisconsin/05/2005, A/Brisbane/07 /2007 and A/Perth/16/2009 as references. In reference 60 HA genes and 55 NA genes, both 44 HA and NA genes viruses were isolated from Asia, Europe, North America, South America and Oceania from 1968 to 2011 while 16 HA genes from Asia (4 genes from Japan and Russia Asia region), Europe (8 genes from Russia and George) and North America (4 genes from USA) and 9 NA genes from Asia (2 genes from Russia Asia region) and Europe (7 genes from Russia and George) isolated in 2012.

**Table 1:** Influenza A H3N2 viruses used in this study

**Strain designation**	**Collection date (month/day/year)**	**Geographical location of isolate**	**Cell line isolated in**	**GenBank sequence accession no.**	
				**HA**	**NA**
A/Guangdong/174/2011	8/16/2011	Zhuhai	MDCK2	CY125683	CY125684
A/Guangdong/187/2011	9/26/2011	Zhuhai	MDCK2	CY125685	CY125686
A/Guangdong/192/2011	9/15/2011	Dongguan	MDCK2	CY125687	CY125688
A/Guangdong/222/2011	11/23/2011	Shaoguan	MDCK1	CY125689	CY125690
A/Guangdong/947/2012	3/27/2012	Meizhou	MDCK1	CY125691	CY125692
A/Guangdong/1078/2012	4/9/2012	Maoming	MDCK1	CY125677	CY125678
A/Guangdong/1104/2012	3/29/2012	Zhuhai	MDCK1	CY125679	CY125680
A/Guangdong/1154/2012	4/25/2012	Shaoguan	MDCK2	CY125681	CY125682

### Molecular detection of viral genes

Primers specific for the HA and NA genes of human H3N2 isolated from 1968–2010 were designed using Primer Premier 5.0 (Premier Biosoft International, Palo Alto, CA, USA) and synthesized by Life Technologies (Shanghai, China). These primers have been used in a previous study [6]. Viral RNA was extracted using a Qiagen QIAamp Viral RNA mini Kit, and reverse transcription polymerase chain reactions (RT-PCRs) conducted with Qiagen Sensiscript Reverse Transcriptase and Takara PyroBest Taq. Amplicons were purified with a Qiagen Gel Extraction Kit and sequenced with an ABI PRISM BigDye Terminator v3.0 Ready Reaction Cycle Sequence Kit on an ABI PRISM 3100 Genetic Analyzer. The obtained sequences were analyzed using Lasergene 7.1.

### Phylogenetic analysis and evolutionary dynamics

Virus population dynamics over time were estimated using the BEAST package v1.70 [9] and applying Bayesian Markov chain Monte Carlo (MCMC) methods. Phylogenetic trees were generated, and the reliability of trees tested by bootstrap analysis using 1000 replicates. The MCMC analysis was run for 50,000,000 generations, with stationarity and mixing efficiency examined using Tracer.

### Evolutionary selection and N-glycosylation

Single likelihood ancestor counting (SLAC) was appropriate for large alignments but possibly underestimates the number of positively selected sites [10]. The fixed-effects likelihood (FEL) and the internal fixed-effects likelihood (IFEL) took the synonymous and non-synonymous substitutions into account and could be efficiently parallelized. Potential N-glycosylation sites were predicted using NetNGlyc 1.0 server [11]. This software application predicts N-glycosylation sites in human proteins using artificial neural networks that examine the N-X-S/T (Asn-Xaa-Ser/Thr) amino acid sequence [12].

### Antigenic analysis, epitope region and three-dimensional (3D) structure

Cross-reactivity of isolated viruses was investigated using hemagglutination inhibition (HI) assays [13]. The variation sites in H3N2 virus HA and NA gene sequences isolated from 2011 and 2012 were compared with previously identified epitopes of HA and NA proteins [14]. The 3D structures of HA and NA proteins were established using the I-TASSER server [15]. Models were modified with UCSF Chimera1.5.3 [16].

## Results

### Phylogenic diversity and variation

**Figure 2:**
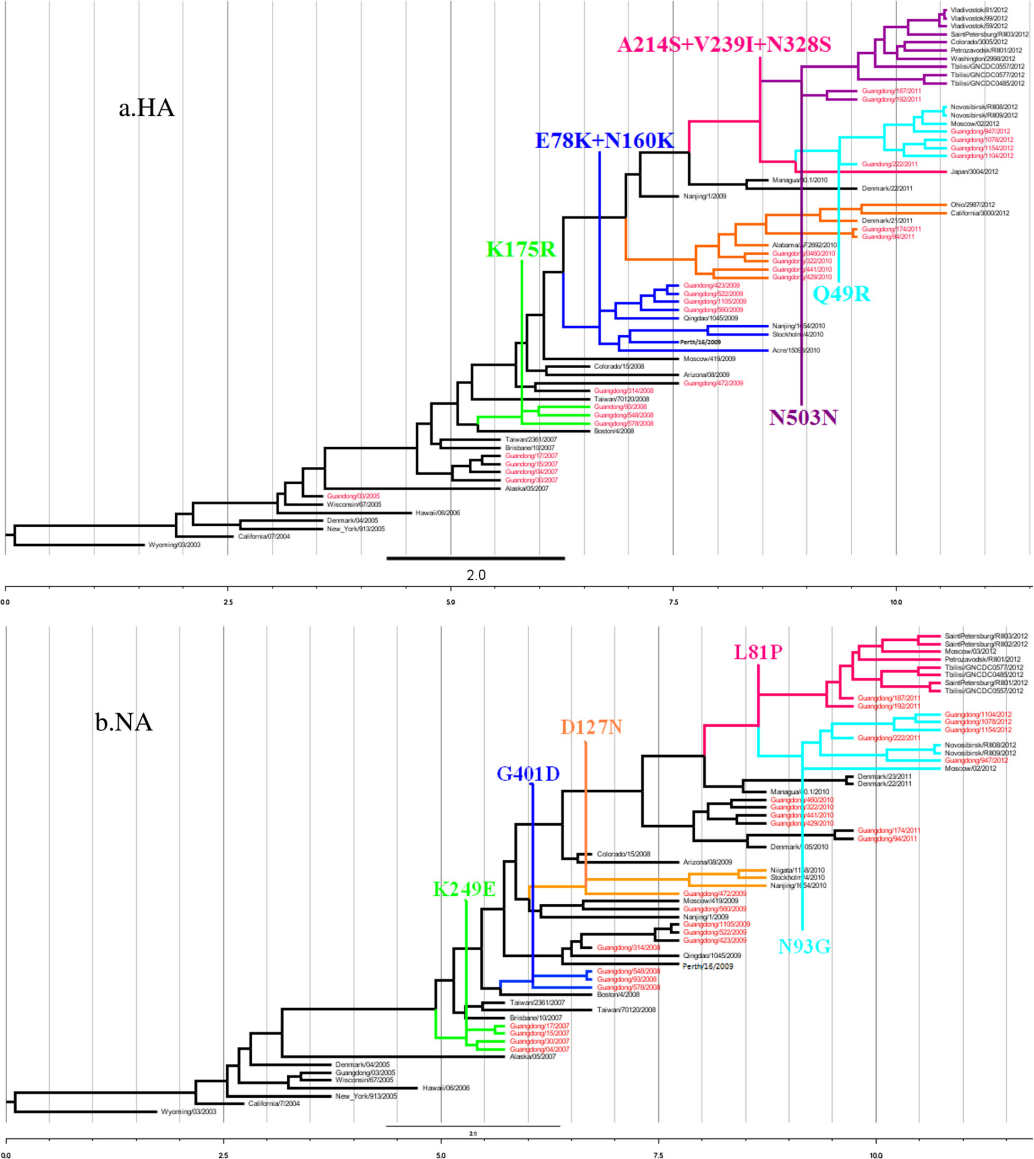
**Phylogenetic analysis of influenza H3N2 HA and NA genes applied MCMC. a.** HA; **b.** NA. The strains shared the same mutation sites clustered into one clad in the same color, where Guangdong strains were in red.

Using five vaccine strains as a reference, amino acid substitution sites in HA genes of Guangdong strains and other strains isolated from 2007–2012 became apparent. These substitutions included K174R in the 2008 Guangdong strains, along with E78K and N160K in A/Perth/16/2009 and the 2009 Guangdong strains. Since 2009, two groups of strains have emerged. The first group includes strains A/GD/441/2010, A/GD/94/2011, and A/California/3000/2012; these contain the HA-D69N, HA-Y110H, HA-I246V and HA-E296A/T substitutions. The second group includes strains A/GD/222/2011, A/GD/187/2011, A/GD/1154, and A/GD/222/2011; these strains contain the HA-A214S, HA-V239I and HA- N328S substitutions (Figure 2a). In two 2011 Guangdong strains (A/GD/94/2011 and A/GD/174/2011) we observed G21E and E296T substitutions in HA, while two USA 2012 strains also contained the E296T substitution. For the Guangdong strain group containing the HA-A214S, V239I and N328S substitutions, we also noticed Q49R and S61N substitutions in A/GD/222/2011 and A/GD/1154/2012; while A/GD/187/2011 and Russian strains from 2012 only contained D503N.

Using the five vaccine strains as a reference, amino acid substitution sites in the NA genes of Guangdong and other strains isolated from 2007–2012 were apparent (Figure 2b). Since 2009, variation sites in strains from around the world have been seen: L81P for 2011 Guangdong isolates, and Russian and Georgian isolates from 2012; D93G for A/GD/222/2011, and Russian and Guangdong isolates from 2012; S367N and K369T for Guangdong isolates from 2010–2012, Danish isolates from 2011, and Russian and Georgian isolates from 2012; N402D for 2011–2012 Guangdong isolates, Japanese and Swedish isolates from 2010, and Russian and Georgian isolates from 2012. In addition, the K249E substitution occurred in four 2007 Guangdong strains, G401D was seen in three 2008 Guangdong strains, and D127N was found in A/GD/472/2009 and three other strains.

### Evolutionary selection and N-glycosylation

Only codon 173 (L/S) of the 566 codons in the HA gene was a positively selected site with a dN-dS of 3.08, and a *P*-value of 0.0944 for SLAC. Codons 237 (P/S/T/L) and 239 (V/I) were positively selected sites with dN-dS of 7.56 × 10^8^ and 6.96 × 10^8^, respectively, and *P*-values of 0.0318 and 0.0794, respectively for FEL. Codons 174 (K/N/R/G) and 209 (S/F) were positively selected sites with normalized dN-dS of 16.7 and 18.6 respectively, and *P*-values of 0.0502 and 0.0315 respectively for IFEL.

Codon 338 (L/R/F), of the 469 codons for NA was a positively selected site with a dN-dS of 9.39 × 10^8^ and a *P*-value of 0.0498 for FEL. Codons 338 and 372 (S/L/F) were positively selected sites with dN-dS of 1.15 × 10^9^ and 1.23 × 10^9^ respectively and *P*-values of 0.0828 and 0.0887 respectively for IFEL.

We identified 13 potential glycosylation sites in the HA gene of the A/GD/1154/2012 isolate: NST_024–026_, NGT_038–040_, NAT_054–056_, NSS_061–063_, NCT_079–081_, NES_138–140_, NWT_142–144_, NGT_149–151_, NNS_160–162_, NVT_181–183_, NST_262–264_, NGS_301–303_ and NGT_499–501_. For the A/Perth/16/2009 HA gene, 11 potential glycosylation sites were present, with only NSS_061–063_ and NNS_160–162_ missing. Using the A/Perth/16/2009 HA gene as a reference, S61N was seen in 2011–2012 Guangdong and other strains, resulting in a gain of the NSS_061–063_ glycosylation site. The N161S substitution occurred in the 2011–2012 Guangdong strains, resulting in an increase of the NNS_160–162_ glycosylation sites. The S140C substitution occurred in A/Tbilisi/GNCDC0557/2012, resulting in deletion of the NES_138–140_ glycosylation site.

There were eight potential glycosylation sites in the NA gene of the A/GD/1154/2012 isolate: NIT_61–63_, NTT_70–72_, NWS_86–88_, NDT_146–148_, NAT_200–202_, NGT_234–236_, NDS_329–331_, and NRS_402–404_. The N402D substitution occurred in the 2011–2012 Guangdong isolates, the 2010 Japanese and Swedish isolates, and the 2012 Russian and Georgian isolates. This resulted in deletion of the NRS_402–404_ glycosylation site.

### Antigenic analysis, epitope and 3-D structure

**Figure 3:**
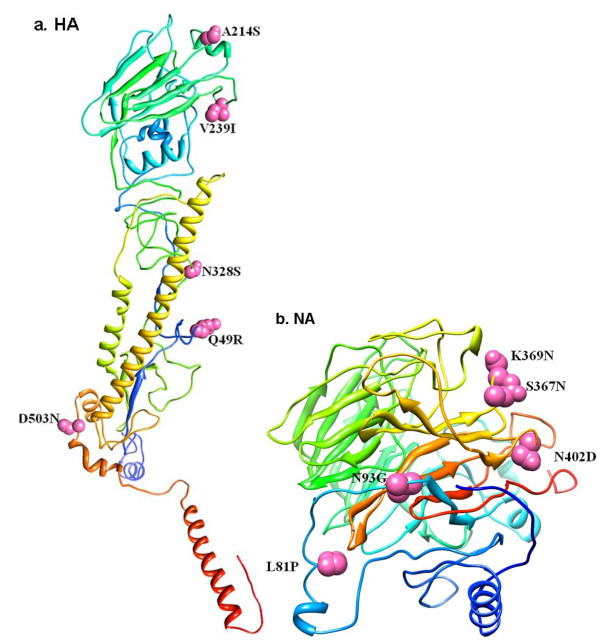
**Mutation sites on HA and NA proteins of H3N2 viruses isolated during 2011-2012.** The 3D structures were modeled from strain A/Guangdong/1154/2012, where the red balls were the mutation sites.

Hemagglutination inhibition assays were conducted using antisera from rabbits infected with Guangdong H3N2 viruses isolated from 2009–2012. When compared with A/GD/1105/2009, strain A/GD/1154/2012 exhibited a 4-fold difference in titers (Table 2). Each mutation site in HA (D69N, Y110H, I246V, E296A/T, A214S, V239I and N328S) involved the epitope regions of A/New York/384/2005 (Table 3). The 3D structures of HA and NA proteins for A/GD/1154/2012 were modeled; HA (Q49R, A214S, V239I, N328S and D503N) and NA (L81P, D/N93G, S367N, K369N and N402D) variation sites were labeled in the 3D structures (Figure 3).

**Table 2:** Hemagglutination inhibition results for the H3N2 strains examined in this study

**Virus**	**Reciprocal HI titer for antiserum**		
	**A/GD/1105/2009**	**A/GD/222/2011**	**A/GD/1154/2012**
A/GD/1105/2009	1:2560	1:640	1:640
A/GD/222/2011	1:1280	1:1280	1:1280
A/GD/1154/2012	1:640	1:640	1:1280

*GD*, Guangdong.

Strain A/GD/1105/2009 is a vaccine strain.

**Table 3:** Sites of variation associated with antigenic epitopess

**Gene**	**Position**	**Mutation**	Epitope ID*
HA	69	D → N	129460, 130279, 128706
HA	110	Y → H	128649, 128428, 129924
HA	246	I → V	129617, 97393, 129652, 97243, 129656
HA	296	E → A/T	129824, 128724
HA	214	A → S	130111, 129953
HA	239	V → I	129617, 97393, 129652
HA	328	N → S	129763, 129381. 129136
NA	81	L → P	130081, 128380
NA	93	D → G	130400, 129643

^*^The reference B cell epitopes/ T cell epitopes of H3N2 strain was from A/New York/384/2005.

## Discussion

Mutated amino acids around human leukocyte antigen (HLA)-associated sites, especially those that are typically conserved, suggest that cooperative interactions act to preserve the local structural stability and protein function when mutations occur that confer evasion of cytotoxic T lymphocytes (CTLs) [17]. For the HA genes of isolated strains, the D69N, Y110H, I246V and E296A/T substitutions occurred around 2010 [6], while A214S, V239I and N328S have been present since 2011. Accumulation of HA (D69N, Y110H, I246V, E296A/T, A214S, V239I and N328S), along with NA (L81P and D93G) mutations, drove antigenic drift possibly giving rise to the H3N2 influenza epidemic in Guangdong and Southern China in 2012. The HA-A214S, -V239I and -N328S mutations occurred in the second half of 2011 (Table 1 and Figure 2). As the 2012 influenza season approached, these variations in circulating strains became prevalent, resulting in local epidemics throughout Southern China.

According to a previous study that focused on isolates from 2011 [18], HA1 genes were sub-divided among the P (A/Perth/16/2009-Clade) and V (A/Victoria/208/2009-Clade) clades. The former included subgroups1 and 2, while the latter included subgroups 3–6. When we compared our findings with the results from Klimov [18] and Huang [6], mutations A/N144T/D and K/N145N/S were only observed in the previous studies; the mutations we have reported here were not evident in Klimov’s work. The mutations in the 2012 Guangdong isolates appear to have given rise to antigenic drift. Generally, five HA1 antigenic regions are analyzed and associated with antigenic drift that results in an epidemic. However, for the particular epidemic we investigated, these five regions were not seemingly associated with antigenic drift. Only the A214S substitution occurred in epitope D and the RBS. Beside the five HA1 antigenic regions, many amino acid sites were unclassified but still considered important [6]. Using the H3N2 HA and NA protein sequences of A/New York/348/ 2005 as a reference, all mutated amino acid sites were related to previously identified epitopes, which might involve in antigenic presentation / recognition / response. This indicated that mutations of B cell epitope / T cell epitope regions in the Immune Database influenced antigenicity to some degree [14]. These B cell epitopes / T cell epitopes span 17–18 amino acids in the A/New York/348/2005 strain. Further research is required to confirm the relationship between antigenicity and mutations in HA and NA genes.

As was observed for of major capsid L1 protein of HPV-16 and −18, amino acids at positions 174 (K/N/R/G), 209 (S/F), 237 (P/S/T/L) and 239 (V/I) in HA, and at positions 338 (L/R/F) and 372 (S/L/F) in NA were positively selected sites according to SLAC, FEL and IFEL (*P* < 0.1), indicating that these sites avoided immunological pressure for their continued persistence [19].

Only 76% of positive scored sequons according to NetNGlyc are modified by N-Glycans with a bias towards Thr-containing sequons [20]. Compared with A/Perth/16/2009, two glycosylation sites (NSS_061–063_ and NNS_160–162_) in HA genes were prevalent in 2011–2012 Guangdong strains, with the NA glycosylation site N_402_D deleted. There are five possible functions for glycosite migration in human influenza viruses [21]: to more effectively mask the antigenic sites; to more effectively protect enzymatic cleavage sites of NA; to stabilize polymeric structures; to regulate receptor binding and catalytic activities; and to balance the binding activity of HA with the release activity of NA. Five HA glycosites and two NA glycosites were positive sites in this study. Gains in N-glycosylation sites were likely to be positively selected for shielding antigenic sites from immune responses [22]. The acquisition of glycosylation at residues 144 of pH1N1 was associated with viral replication, virulence and transmissibility and provided insights into the evolution dynamics of influenza viruses with implications in vaccine immunogenicity [23]. The loss of a glycosylation site might also be important in antibody recognition [22], where a deletion of the NA-NRS_402–404_ occurred in the 2011–2012 Guangdong and other isolates in this study. With respect to HA/NA N-glycosylation changing, further analysis is required to determine any relationship between their structure mutation and function influence. At the antigenic sites of HA protein, positive selection appeared to have effected radical and conservation substitution in term of the charge of the amino acids, suggesting that antigenic drift is not a byproduct of HA evolution in H3N2 viruses [22].

Kenyan H3N2 viruses isolated during 2006–2007 revealed unique genetic variations, with several amino acid substitutions located at immunodominant epitopes of the HA1 protein [24]. These mutations included V112I at site E, K173 E at site D and N278K at site C. These mutations possibly result in a conformational change to the HA molecule, thereby exposing novel epitopes and thus abrogating the binding of pre-existing antibodies at these sites. A Canadian study in 2011 involving antigenic and molecular characterization of H3N2 viruses over three seasons revealed that the number of HA mutations was important, along with the nature and location of key mutations, and played a significant role in antigenic drift [5]. From our findings, presented in this report, the 2012 strains had evolved genetically and antigenically from the A/Perth/16/2009 vaccine-like strains. The A/GD/1154/2012 strain was antigenically distinct from the A/GD/1105/2009 strain, suggesting that it may be the parental strain responsible for the 2012 H3N2 influenza virus epidemics in Southern China. Although influenza H3N2 viruses varied genetically and antigenically during 2009–2011 [6], we concluded that antigenic drift in 2012 along with the accumulation of evolutionary mutations in viruses isolated in 2012 played an important role in the influenza epidemics of Guangdong and Southern China during that period.

## Competing interests

None of the authors has a financial or personal competing interest related to this study.

## Authors’ contributions

PH and SYY conceived and designed the study. JZ, LJL, PH, XLZ, LRZ, XZ, YHZ and HZN were involved in sample collection, virus isolation and genetic analysis. JZ, LJL, PH and JY contributed to data analysis and writing the manuscript. All authors have read and approved the final version of this manuscript.
